# Why is the inpatient cost of dying increasing in India?

**DOI:** 10.1371/journal.pone.0203454

**Published:** 2018-09-10

**Authors:** Sumit Kumar Das, Laishram Ladusingh

**Affiliations:** 1 Biostatistics and Epidemiology, International Institute for Population Sciences, Deonar, Mumbai; 2 Department of Mathematical Demography and Statistics, International Institute for Population Sciences, Deonar, Mumbai; Dr. Rajendera Prasad Government Medical College, INDIA

## Abstract

**Introduction:**

There is an evidence of increasing inpatient expenditure for decedents. Estimates used to assess the economic burden of out-of-pocket (OOP) healthcare expenditure provide an underestimation for inpatient decedent cases. The aims of this paper are to study the trend and pattern of inpatient decedent expenditure and decipher the reasons behind the increasing cost in India.

**Methods:**

Using three rounds of national level National Sample Survey (NSS) data on morbidity & healthcare conducted during 1995–2015 in India, total and component-wise cost of dying was estimated by the socio-demographic characteristics and types of diseases. Generalised linear model was employed to find the changing effect of inpatient decedents on inpatient expenditure on three-time points.

**Results:**

More than half among inpatient decedents were elderly. Mean inpatient expenditure for neoplasm, circulatory system-related diseases and external causes of mortality and morbidity increased substantially during these two decades. Mean decedent inpatient expenditure become double, diagnostic and bed charges increased by 243%, 323% respectively during 2004–05 to 2014–15. During 2014–15 average decedents aged 15–59 years spent ₹53599 in last twelve month of their life. Controlling all other potential factors, the inpatient expenditure among decedents increased substantially between 1995–96 and 2014–15.

**Discussion:**

Out-of-pocket inpatient health expenditure widened between survivor and decedents in between 1995–2014. Increase in the proportion of elderly, proportion of non-communicable and lifestyle-related diseases, expenses on drugs, diagnostics, bed charges largely private sector expenses were the leading reasons for increasing inpatient decedent expenditure. Age-based risk adjustment and modification of end-of-life care are strongly required, future social insurance based on the health-based value of out-of-pocket expenditure rather than their pure consumption value need to be designed to tackle the burden.

## Introduction

Catastrophic health expenditure [1,2] and impoverishment [3] is often used for assessment of economic burden of out of pocket (OOP) healthcare expenditure. These measures underestimate the economic burden of OOP for households whose members have died during the course of treatment. At the macro level from the perspective of public health financing ignoring the differential in OOP healthcare expenditure between survivors and decedents shall end up with estimates far short of the required budget. Financing end of life care in the USA is estimated to have spent 10–12% of all healthcare spending [4] and end of life care is provisioned in Medicare, the largest health insurance plan in the USA. Hogan et al. [5] have highlighted that about a quarter of the total Medicare budget was spent on healthcare of beneficiaries in their last year of life and further Lubitz & Riley [6] have added that 40% of it was spent in the last 30 days. Seshamani & Gray [7] and Zweifel et al. [8] have used econometric models to confirm the hypothesis that both age and proximity to death are the significant determinants of healthcare expenditure. However, in India, there is no evidence of provisioning of the end of life care and perhaps this has resulted in low public health spending. This is partly due to the lack of data on medical history and OOP expenditure for each episode of ailments treated as outpatient or inpatient and secondly as a result of ignorance of the high OOP expenditure at the end of life. Among the first study in the Indian context, Ladusingh & Pandey [9] have provided empirical evidence using from the National Sample Survey Organisation (NSSO) 60th round (2004) data that throughout the lifecycle inpatient healthcare OOP expenditure of decedents is much higher than that of survivors. Mohanty et al. [10] have highlighted that during 1993–2012 the increase in annual per capita health expenditure is twice the annual growth rate of per capita consumption expenditure but it ignored to take account of OOP expenditure of decedents. The Availability of recent NSSO 71st round (2014–15) data has provided an opportunity to re-visit the gap in OOP inpatient healthcare expenditure between decedents and survivors and answer questions such as, whether the gap in OOP inpatient healthcare expenditure between decedents and survivors has widened? What contributes to the increasing cost of inpatient death? Unfolding answers to these questions would facilitate to comprehend more realistically the hidden escalating economic burden of OOP inpatient healthcare expenditure. The study is expected to provide key inputs for strengthening the health system in India to meet the pressing demand for public healthcare and cope with the epidemiological transition.

The paper is organized as follows: data and methods are described in the next section, followed by a section on results and ends with a section on discussion.

## Data and methods

### Data

Ministry of Statistics and Programme Implementations (MOSPI) of the Government of India (GOI) collects data on morbidity and health care in India for every ten years interval on a nationally representative sample of households. National Sample Survey Organisation (NSSO), a wing of MOSPI, is responsible for conducting large-scale sample surveys in the diverse field on all India basis. The datasets are de-identified before bringing into the public domain. Unit-record data from last three NSS rounds on ‘Morbidity & Health Care’; the 52^nd^ round (1995–96)[11], 60^th^ round (2004–05) [12] and 71^st^ round (2014–15) [13] were used to study the change in the inpatient decedents expenditure. NSS 52^nd^ round was a full year survey done in four sub-rounds (July 1995-June 1996), while, NSS 60^th^ and NSS 71^st^ rounds was a half year survey done in two sub-rounds (January 2014-June 2014). To check the robustness, the 52^nd^ round was divided into two half (January 1996-June 1996, July 1995-June 1996) and compared the mean inpatient expenditure. The estimated values were similar in both the half with 95 per cent confidence level. The sampling designs adopted in three rounds of NSSO surveys were multi-stage stratified sampling and were comparable. These surveys were carried over the entire country excluding few places. Detailed information on the expenditure incurred on each episode of hospitalisation within 365-days there in all the. Individuals found in the sampled household were 629888, 383338 and 333104 respectively for the three rounds. And out of those households, the numbers of deaths occurred in the last 365 days were 3520, 1717 and 2395 respectively for three-time points. Reported diseases and out of pocket expenditure for one year reference period for hospitalization cases were considered for this study. Healthcare expenditure were for clinical and pathological check-ups, doctor’s fee, medicine, diagnostic charges, bed charges, others medical expenses (attendant charges, physiotherapy, personal medical appliances, blood, oxygen etc.), and non-medical expenditure includes transport for inpatient and for other household members, their food, expenditure on escort and lodging charges if any. There were few cases for which complete information was absent and these were excluded in the analysis. No selectivity in missing was noted and not included in the analysis. After excluding unmatched diseases and dropping missing values total numbers of inpatient cases used for the analysis in 1995–96, 2004–05 and 2014–15 were 23228, 30665 and 40191, out of which 5.97, 2.18 and 5.70 per cents cases respectively died while undergoing treatments. The analyses were performed applying sampling weight given in the dataset.

### Methods

All out of pocket (OOP) health care expenditures in the year 1995–96 and 2004–05 were adjusted to the price of 2014–15 using Net State Domestic Product (NSDP). According to Reserve Bank of India (RBI), in monetary terms, the NSDP is defined as a measure of the volume of all goods and services produced within the boundaries of the state during a given period of time after deducting the wear and tear of depreciation, accounted without duplication [14]. State level data of NSDP, which is calculated by removing the consumption of fixed capital from gross state domestic product for each sector, at the current price and constant price, were collected from Reserve Bank of India (RBI) website of the government of India [14, 15]. The ratios between NSDP at the current price and at the constant price were calculated for 2014–15 for every state in India. These ratios were used as an inflator/deflator, which is multiplied by the expenditure incurred by each inpatient cases state wise. For a long-term trend study, the choice of appropriate base year is an essential issue. It is practically correct to take a base year somewhere in the middle of the series [16]. It does not seem to be appropriate to take two different base years (i.e. 1995–96, 2004–05) because the goods composition and the relevance of the products and services may be changing over time. Hence, the year 2004–05 was used as a base year for this analysis covering the period of 1995–96 to 2014–15. In other studies, Gross Domestic Product (GDP) [17], Consumer Price Index (CPI) [18] were used as a deflator to compare the expenditure in different rounds, but those deflators are at national level, state level variation is not captured. On the other hand, NSDP equal to the GDP minus depreciation on capital goods at the state level. If the constant price is taken in the middle of the series it automatically adjust the discounting effect.

Variables used in this study were inpatient expenditure (medical & non-medical), survival status of inpatient, age of the inpatient cases (less than 15, 15–59, 59 & more), length of hospital stay (within one week, within one month), ailment of hospitalisation, types of hospital (public, private). Diseases are classified into thirteen broad categories based on International Classification of Diseases (ICD-10), which is a standard diagnostic tool for epidemiology, health management and clinical purposes [19]. Descriptive statistics, bivariate analysis, and diagrammatic representations were used to describe the change in the mean inpatient expenditure for the decedent and survivors. To explore the effect of inpatient decedents on overall inpatient expenditure adjusting all other factors three separate regressions were applied on three-time points. The outcome variable (i.e. overall inpatient expenditure) used in this study is usually non-parametric and positively skewed with influential outliers. The use of traditional Ordinary Least Square (OLS) with log-transformation cannot correctly capture the skewness of the data. Generalised Linear Model (GLM)[20] can flexible handles the skewed datasets and reduce the problem of outcome transformation. Here, in this study GLM with gamma distribution and log-link function were applied to examine the change in the effect of survival status on inpatient expenditure controlling all other factors.

## Results

Table 1 presents the change in the percentage distribution and mean expenditure of inpatient cases by demographic and hospital characteristics over three-time points i.e., 1995–96, 2004–05 and 2014–15. In 1995–96, out of total inpatient decedents, 19.2 per cent were aged less than 15 years, which was drastically reduced to 6.6 per cent in the year 2014–15. Deaths in between 15–59 years among the inpatient cases were declined from 46.5 per cent to 37.2 per cent during 1995–96 to 2014–15. The significant increment was observed in the age group 60 & more in the inpatient dying in between 1995–96 (34.4 per cent) and 2014–15 (56.3 per cent). In the year 1995–96, among the total hospitalised death cases 53.5 per cent spent less than one week at the hospital. And approximately 89.8 per cent of total inpatient decedent cases spent less than one month at hospitals in 1995–96. The percentage of death among inpatient decedents within one week increased by 10.2 units whereas the percentage of deaths within one-month increased by 5.6 units in the year 2014–15. The estimated mean inpatient expenditure for the age group less than 15 years increased by ₹8377 and ₹24946 for survivors and decedents respectively in between 1995–96 and 2014–15. The maximum increment of mean inpatient expenditure (₹37426) was observed among inpatient decedents of 15 to 59 years age group. Whereas, mean inpatient expenditure increased by ₹10986 among survivors aged 15 to 59 years in between 1995–96 and 2014–15. In public hospitals mean inpatient expenditure for decedents increased by ₹8503 during 1995–96 to 2014–15. The mean inpatient expenditure among survivors in private hospitals was found to have increased by ₹14172 during 1995–96 to 2014–15. Whereas, in the private sector, the mean inpatient expenditure among decedents were found to have increased ₹43505 in between 1995–96 and 2014–15. The mean inpatient expenditure among decedents h increased across all wealth status while increment in the mean inpatient expenditure of survivors was observed in highest wealth tertile. The mean inpatient expenditure among decedents increased ₹26203, ₹22670 and ₹49839 for poor, middle and rich respectively during 1995–96 to 2014–15. Chi-square test of association, independent t-test and analysis of variance test were performed and all the groups were found to be associated with survival status at significant level P<0.05 in all the three time points.

**Table 1:** Distribution of inpatients by demographic characteristics, hospital type, length of hospital stay, survival status and mean out-of-pocket expenditure in India, 1995–96, 2004–05 and 2014–15.

		Survivors			Decedents			Survivors			Decedents		
		1995–96	2004–05	2014–15	1995–96	2004–05	2014–15	1995–96	2004–05	2014–15	1995–96	2004–05	2014–15
		N = 21842	N = 29997	N = 37888	N = 1386	N = 668	N = 2303	Mean inpatient expenditure (in Rupees)					
	Overall	n(%)	n(%)	n(%)	n(%)	n(%)	n(%)	7385	15485	19449	13313	22649	43897
Age group	less than 15	4500(20.8)	5554(19.2)	6677(16.5)	217(19.2)	83(18.1)	167(6.6)	3534	8306	11897	7951	12775	32897
	15–59	13996(62.7)	19191(62.5)	22930(60.4)	706(46.5)	324(45.6)	1024(37.2)	8604	16848	19594	16173	25288	53599
	60 and more	3346(16.5)	5252(18.3)	8281(23.1)	463(34.4)	261(36.3)	1112(56.3)	7600	18374	24469	12431	24252	38751
Wealth Status	Poor	4500(22.4)	10116(32.7)	11342(29.2)	235(15.1)	226(39.4)	676(29.5)	2420	9921	12063	1654	14161	29286
	Middle	7384(35.5)	10240(35.8)	12326(33.0)	390(34.9)	233(32.2)	704(34.5)	3744	13242	15165	5346	20181	28032
	Rich	9957(42.1)	9641(31.5)	14215(37.8)	761(49.9)	209(28.4)	923(36.0)	13102	23793	28884	22415	37247	70886
Type of hospital	Public	11966(48.4)	13651(39.8)	16089(37.2)	871(57.2)	369(50.5)	1112(44.9)	4388	8325	7361	9548	14043	18690
	Private	9687(51.6)	16346(60.3)	21799(62.8)	510(42.8)	299(49.5)	1191(55.1)	10235	20208	26563	18357	31425	64127
Length of Hospital Stay	Less than one week	12381(59.2)	18548(64.9)	27891(74.1)	705(53.5)	357(53.9)	1399(63.7)	3276	8003	11797	5733	12991	16662
	Less than one month	20623(95.2)	29019(97.3)	37294(98.5)	1253(89.8)	623(93.8)	2182(95.4)	6633	14006	18253	12178	20541	37697

**Fig 1:**
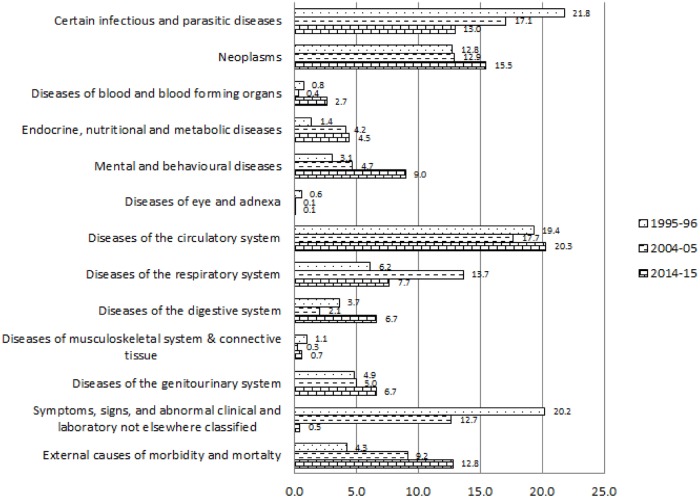
Causes of hospitalisation among inpatient death cases classified by ICD10 during 1995–96 to 2014–15, India.

**Fig 2:**
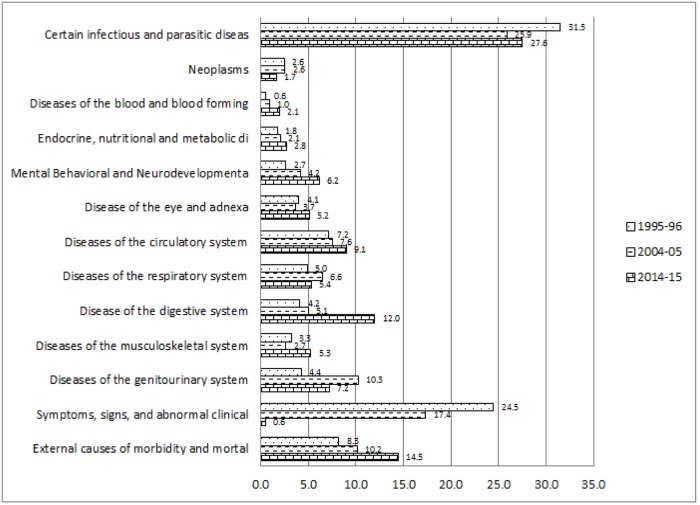
Causes of hospitalisation among inpatient survivors classified by ICD10 during 1995–96 to 2014–15, India.

**Fig 3:**
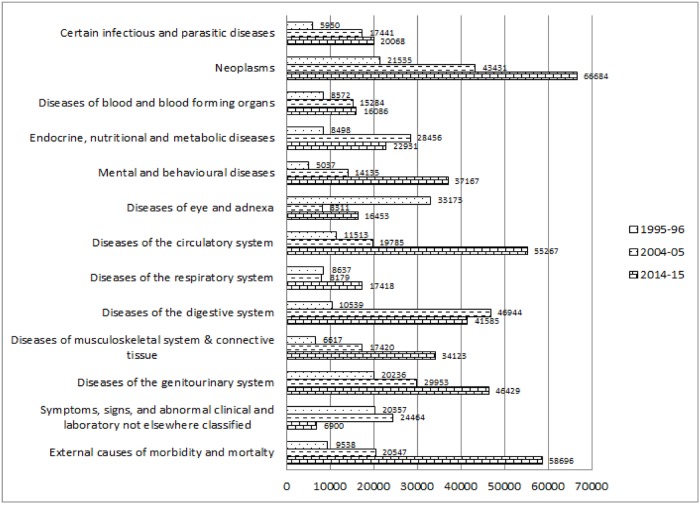
Disease-specific expenditure of inpatient decedents classified by to ICD10 (in Rupees) during 1995–96 to 2014–15, India.

Fig 1 explains the change in the percentage of causes of hospitalisations among inpatient deaths classified by ICD 10. The percentage among the inpatient deaths suffering from certain infectious and parasitic diseases declined from 21.8 per cent in 1995–96 to 13 per cent in 2014–15. Percentage of inpatient deaths due to neoplasm increased by 2.7 units during 1995–96 to 2014–15. Likewise, the percentage of hospitalisation among inpatient death cases because of mental and behavioural diseases considerably increased from 3.1 per cent to 9 per cent during 1995–96 and 2014–15. Similarly, Percentage of deaths among inpatients because of diseases related to blood and blood-forming organs; diseases related to Endocrine, nutritional and metabolic; diseases related to the genito-urinary system significantly increased during 1995–96 to 2014–15. The percentage of diseases, which were found to be abnormal in laboratory findings, reduced drastically from 20.2 per cent to 0.5 per cent respectively among the inpatient decedent cases in between 1995–96 and 2014–15. However, the percentage of inpatient death due to external causes of morbidity and mortality increased by 8.5 units during 1995–96 to 2014–15. Fig 2 depicts the per cent distribution of causes of hospitalisation among inpatient survivors classified by ICD 10 during the year 1995–96, 2004–05 and 2014–15. Percentage of hospitalisation among inpatient survivors due to certain infectious and parasitic diseases reduced from 31.5 per cent to 27.6 per cent in between 1995–96 and 2014–15. The percentages of hospitalisations among the inpatient survivors because of diseases related to digestive system increased from 4.2 per cent to 12 per cent during 1995–96 to 2014–15. Whereas, external causes of morbidity and mortality increased by 6.2 units as a reason of hospitalisation among inpatient survivors in between 1995–96 and 2014–15. Diseases of blood and blood-forming organ; endocrine, nutritional and metabolic diseases; mental and neurodevelopmental diseases; diseases of eye and adnexa; diseases of circulatory system; diseases of musculoskeletal system increased significantly among the reasons behind hospitalisation of inpatient survivors. Unidentified symptoms of hospitalisations among survivors considerably declined from 24.5 per cent to 0.6 per cent in between 1995–96 and 2014–15. Fig 3 shows the disease-specific expenditure of inpatient decedent cases classified by ICD 10 for the year 1995–96, 2004–05 and 2014–15. The mean expenditure among the decedent’s hospitalization cases for certain infectious and parasitic diseases was ₹5950 in the year 1995–96 which increased to ₹20068 in the year 2014–15. The substantial increment was observed during 1995–96 to 2014–15 in the mean inpatient decedent expenditure (₹45149) of neoplasm related diseases. Mean inpatient expenditure of disease related to blood and blood-forming organs, mental and behavioural diseases, diseases related to circulatory system, diseases related to musculoskeletal system and disease related to genitourinary system was found to have increased significantly. The expenditure increased by ₹7514, ₹32130, ₹43754, ₹27506 and ₹26193 respectively during 1995–96 to 2014–15.

**Fig 4:**
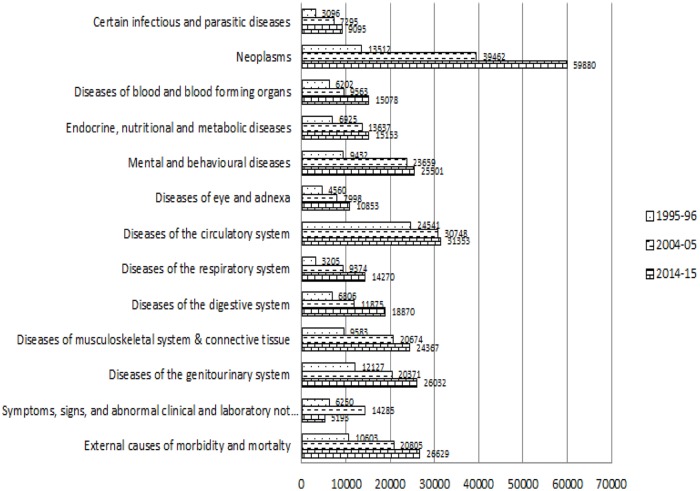
Disease-specific expenditure of inpatient survivors classified by ICD 10 (in Rupee) during 1995–96 to 2014–15, India.

The mean inpatient expenditure due to disease related to respiratory system among the inpatient decedent cases was ₹3658 in the year 1995–96; it increased to ₹17418 during 2014–15. The considerable increment was observed in the mean inpatient expenditure is observed in external causes of morbidity and mortality related cases, the expenditure increased from ₹9538 to ₹58696 in between 1995–96 and 2014–15. Fig 4 represents the trend of disease-specific mean inpatient expenditure among survivors classified by ICD 10 during 1995–96 to 2014–15. The significant increment was observed in mean inpatient expenditure among the survivors with neoplasm related diseases, increased by ₹46368 during 1995–96 to 2014–15. Mean inpatient expenditure for survivors due to mental and behavioural diseases was ₹9432 in the year 1995–96, which was found to have increased by ₹25501 in 2014–15. Mean inpatient expenditure for survivors because of infectious and parasitic, blood, endocrine, nutritional, metabolic, eye, circulatory system, respiratory system, digestive system, genitourinary related diseases and external causes of morbidity and mortality was found to have increased in between 1995–96 and 2014–15.

Table 2 depicts the component-wise mean inpatient expenditure (in rupee) and the ratio of expenditure between the year 2004–05 and 2014–15. The decedent mean inpatient increased by 94 per cent and among survivors, it increased by 26 per cent in between 2004–05 and 2014–15. The mean expenses for diagnostic test and bed charges among inpatient decedents increased by 243 per cent and 323 per cent respectively and among survivors, it increased by 59 per cent and 81 per cent respectively during 2004–05 to 2014–15. Mean expenses on medicine (262 per cent), diagnostic charges (463 per cent) and bed charges (207 per cent) among inpatient decedents aged less than 15 years inpatient decedent cases increased significantly. 425 per cent increment was observed in the mean bed charges among decedents aged 15 to 59 years during 2004–05 to 2014–15. Whereas mean diagnostic charges and medicine expenses were found to have increased 299 per cent and 110 per cent respectively among the inpatient decedents aged 15 to 59 years. The mean bed charges and diagnostic charges were found to have increased 189 per cent and 250 per cent respectively among the inpatient decedents aged 60 years and more during the year 2004–05 to 2014–15. Whereas, among survivors, it was recorded at 88 per cent and 124 per cent increment respectively.

**Table 2:** Component-wise mean inpatient expenditure (in rupee) and their ratio between the year 2004–05 and 2014–15, India.

	2004–05		2014–15		Ratio between 2004–05 and 2014–15	
	Decedent	Survivor	Decedent	Survivor	Decedent	Survivor
	Total(₹)	Total(₹)	Total(₹)	Total(₹)	Ratio(%)	Ratio(%)
**All age groups**						
Overall	22649	15485	43897	19438	94	26
Doctor	7673	4262	12962	5193	69	22
Medicine	9404	4644	14543	5307	55	14
Diagnostic	1992	1552	6842	2471	243	59
Bed charges	2103	1709	8892	3087	323	81
Other Medical	4295	3314	5515	2532	28	-24
Transportation	1525	656	1440	675	-6	3
Other Non-Medical	2795	1366	2492	1483	-11	8
**less than 15**						
Overall	12775	8306	32897	11911	158	43
Doctor	3008	1779	7241	3089	141	74
Medicine	3769	2776	13638	3319	262	20
Diagnostic	1472	884	8283	1524	463	72
Bed charges	1676	1260	5146	2381	207	89
Other Medical	3329	2041	7072	1277	112	-37
Transportation	543	415	1349	484	148	17
Other Non-Medical	1084	896	2528	1246	133	39
**15–59**						
Overall	25288	16848	53599	19590	112	16
Doctor	7816	4946	14108	5234	81	6
Medicine	9642	5154	20259	5463	110	6
Diagnostic	2046	1680	8158	2475	299	47
Bed charges	2162	1821	11349	2957	425	62
Other Medical	4729	3577	6965	2324	47	-35
Transportation	1201	727	1832	703	52	-3
Other Non-Medical	2608	1469	3216	1498	23	2
**60 and more**						
Overall	24252	18374	38751	24450	60	33
Doctor	8731	4649	13023	6775	49	46
Medicine	10509	5010	11010	6473	5	29
Diagnostic	2008	1696	5810	3188	189	88
Bed charges	2273	1793	7965	4010	250	124
Other Medical	4172	3682	4367	3991	5	8
Transportation	2427	660	1169	737	-52	12
Other Non-Medical	3761	1488	1992	1614	-47	8

The results of the multivariable analysis are presented in Table 3 for three years 1995–96, 2004–05 and 2014–15. Controlling types of hospital, age of the inpatient cases, types diseases, wealth status of the household of the hospitalised cases; the inpatient expenditure was significantly higher among decedents cases in the year 2014–15 (log(β) = 0.495, P<0.001) as compared to the year 1995–96 (log(β) = 0.427, P<0.001) and the year 2004–05 (log(β) = 0.489, P<0.001). The inpatient expenditure at private hospitals was significantly higher in the year 2014–15 (log(β) = 1.188, P<0.001) as compared to 1995–96 (log(β) = 0.834, P<0.001) and 2004–05 (log(β) = 0.689, P<0.001). After controlling all other factors the inpatient expenditure was found to be statistically significantly higher due to diseases related to respiratory system, diseases of digestive system in the year 2014–15 as compared to 1995–96 and 2004–05. Whereas, the inpatient expenditure was found to be statistically significantly lower due to endocrine, nutritional and metabolic, genitourinary related diseases in the year 2014–15 as compared to 1995–96 and 2004–05. As per as wealth status of the household of inpatient cases is concerned, the expenditure was found to be lower among the inpatient decedent cases middle and highest tertile during the year 2014–15 (log(β) = 0.075, P<0.01; log(β) = 0.462, P<0.001) as compared to the year 1995–96 (log(β) = 0.327, P<0.001; log(β) = 1.061, P<0.001) and 2004–05 (log(β) = 0.162, P<0.001; log(β) = 0.531, P<0.001). The amount of inpatient expenditure for staying more than one week in hospital is significantly higher in the year 2014–15 (log(β) = 1.170, P<0.001) as compared to 1995–96 (log(β) = 1.115, P<0.001) and 2004–05 (log(β) = 1.155, P<0.001).

**Table 3:** Factor associated with inpatient expenditure among individual in 1995–96, 2004–05 and 2014–15.

		1995–96		2004–05		2014–15	
Explanatory Variables	Categories	Log(β)	P value	Log(β)	P value	Log(β)	P value
Survival Status	Survivors	Ref		Ref		Ref	
	Decedents	0.427	0.000	0.489	0.000	0.495	0.000
Type of hospital	Public	Ref		Ref		Ref	
	Private	0.689	0.000	0.834	0.000	1.188	0.000
Age group	Less than 15 years	Ref		Ref		Ref	
	15–59 years	0.250	0.000	0.174	0.000	0.206	0.000
	60 & more years	0.212	0.001	0.149	0.000	0.175	0.000
Diseases type	Certain infectious and parasitic diseases	Ref		Ref		Ref	
	Neoplasm	0.941	0.000	1.366	0.000	1.284	0.000
	Diseases of the blood and blood forming organ	0.186	0.491	0.455	0.000	0.430	0.000
	Endocrine, nutritional and metabolic diseases	0.485	0.001	0.375	0.000	0.282	0.000
	Mental, behavioral and neurodevelopmental diseases	0.572	0.000	0.754	0.000	0.622	0.000
	Disease of the eye and adnexa	0.164	0.113	0.250	0.000	0.183	0.000
	Diseases of the circulatory system	0.726	0.000	1.035	0.000	0.982	0.000
	Diseases of the respiratory system	0.014	0.883	0.149	0.001	0.220	0.000
	Disease of the digestive system	0.410	0.000	0.419	0.000	0.446	0.000
	Diseases of the musculoskeletal system	0.602	0.000	0.671	0.000	0.603	0.000
	Diseases of the genitourinary system	0.707	0.000	0.665	0.000	0.662	0.000
	Symptoms, signs, and abnormal clinical	0.351	0.000	0.466	0.000	0.287	0.041
	External causes of morbidity and mortality	0.595	0.000	0.760	0.000	0.706	0.000
Wealth status	Poor	Ref		Ref		Ref	
	Middle	0.327	0.000	0.162	0.000	0.075	0.001
	Rich	1.061	0.000	0.531	0.000	0.462	0.000
Length of hospital stay	Within one week	Ref		Ref		Ref	
	More than one week	1.115	0.000	1.155	0.000	1.170	0.000
Constant		6.598	0.000	7.631	0.000	7.655	0.000

## Discussion

An underestimation for inpatient decedent cases is obtained while assessing the economic burden of out-of-pocket (OOP) healthcare expenditure. For policy and planning purposes, an understanding of the direction of expenditure among inpatient decedents is required in order to provide important information for healthcare planning. This present study is a first ever attempt in India to find out the trend and pattern across time points and uncovering the reasons behind the unprecedented increment of inpatient decedents expenditure at a disaggregated level. Three rounds of nationally representative survey data were used to boil down under mentioned salient findings.

First, the proportion of elderly (60 & more years) among the inpatient decedents has increased by 21.9 units in between 1995–96 and 2014–15. Second, death related to non-communicable and lifestyle-related diseases are increasing among inpatient decedents cases. Third, the significant increase in Inpatient decedents expenditure in the middle age group. Fourth, among the inpatient decedents; diseases related to neoplasms, circulatory system, genitourinary and external causes of morbidity were found to have a high amount of expenditure for treatment care and many of these are incurable. Fifth, During 2004–05 and 2014–15 the mean inpatient decedent expenditure increased by 94% while it was 26% among survivors. Sixth, the expenditure in private hospital among inpatient decedent cases is significantly higher. Seventh, the mean inpatient expenditure for decedents is almost two times higher than survivors in 2014–15. And finally, controlling all other potential correlates, the inpatient decedent expenditure was found to increase significantly and the difference in the mean out-of-pocket inpatient expenditure between survivors and decedents widened over the period of time.

Along with demographic transition, the epidemiological transition is serving to shift the disease pattern from infectious and communicable diseases to non-communicable chronic diseases among middle and older ages. Disease related to neoplasm, circulatory system and external causes of morbidity and mortality are the principal reasons of hospitalisation among inpatient decedents. Whereas, diseases related to certain infections and parasitic diseases are the prime reasons among inpatient cases who survived. The significant reduction in the percentage of deaths among inpatients with unidentified symptoms indicates the medical advancement and more concentration of certain types of known diseases among all hospitalised cases. It is apparent that the cost of inpatient care tend to increase with age as it was found that expenditure in the age group 15–59 and 60 plus is higher as compared to below 15 age group. This result supports the finding that inpatient decedents expenditure is inversely proportional to the life expectancy [21]. The old, as well as middle age group, bears a significant contribution to this rise for both survivors and decedents [22, 23]. As an evidence of the end of life hypothesis [9], the expenditure for inpatient decedents is one of the most important factors in escalating the expenses for treatment care for non-communicable diseases [24, 25] moreover epidemiological transition [26].

The evidences are there that decedents spending shorter time at hospital tend to spend more as compared to survivors [27, 28]. This increasing percentage of stay among the inpatient decedents is another reason for increment in the cost of dying. This finding indicates the requirement of major improvements in the short stay units (SSU) among the hospitals.

The mean inpatient expenditure has substantially increased among the highest tertile income group in between 1995–96 and 2014–15. Undoubtedly, the private healthcare sector in India provides high quality and adequate services but to avail that service people are compelled to pay the higher sum of wealth. People with more severe and deadly diseases are more likely to go private sector for better treatment, as a result, they pay a substantially higher amount of money than that of the public sector. The gap in expenditure between private and public hospitals has increased over time especially among the older ages [29]. These results are consistent with all other studies in India [30, 31]. The increasing use of private hospitals over two decades in India is one of the reasons for concern while we talk about equity. Although, a limited amount of funds allocated to public health programs, the unwillingness, and inability to shoulder the responsibility led to the dominance of private sector in delivery, out-of-pocket financing, and fee-for-service [32, 33, 34].

It was found from the bivariate analysis of this study that the increment in mean inpatient expenditure was more during the last decade. For further research the total inpatient medical expenditure was broken down into its components out of which medicine expenditure, doctor’s charges and diagnostic charges contributed the maximum. This result indicates the technological development and invention of sophisticated medical equipment in medical care [35, 36]. The term ‘mediflation’ [37] articulated is properly suited here. The mean inpatient expenditure on medicine, bed charges and diagnostic tests have substantially increased among all the age groups. Latimer et. al. [38] has mentioned in his study that patient near to death are more vulnerable to accessing hospital visits, consultations and undergo the major diagnostic test. Buntin et. al. [39] has found that the major reasons behind increasing intensity of physician services are measurable changes in the demographic composition, place of residence, prevalence of health conditions. Promoting generic drugs, reference pricing, incentivizing physicians and pharmacists, long-term value based and outcome-based pricing, public-private partnership can be effective ways for reducing drugs and diagnostic related expenditure.

The results of the study for the period of 1995–2015 suggest that out-of-pocket expenditure near the end of life is large and escalating. This finding of the study shed a light on how much medical related expenditure of dying affect the resource in the last one year of life [40]. This issue has a significant policy concern when we talk about health care reforms. More importantly, the more amount of saving is required in the middle age group and the elderly to cover their future health care needs. Strong rules and regulations are required to chain the over pricing in the private sector. Stringent interventions are required to take from the state as well as central government side with allocated funds as well as monitoring and supervising entire public health care system. The amendment in the existing retrospective risk-sharing arrangement as mentioned by Shmueli et. al. [41] and vliet et. al. [42] is very much relevant in the context of India. The maximum mean inpatient decedent expenditure in the age group of 15 to 59 and 60 plus indicates that there is a strong need for age-based risk adjustment and segmentation of end-of-life care. Insurance indirectly provides protection to the household from large health expenditure. India’s most recent moves towards achieving universal health coverage are to prioritize financial protection health security against impoverishment across all irrespective of socio-economic status [43]. One option to avert high level of expenditure due to inpatient out of pocket expenditure among decedents as well as survivors can be mutual contributions from central and state government as well as from households. This study indicates to design future social insurance based on the health-based value of out-of-pocket expenditure rather than their pure consumption value to tackle the burden.

This study not only fills a research gap in understanding the trend and pattern of inpatient decedent expenditure in the Indian context but also initiates the discussion on the need to rationalize the above-mentioned issue in the framework the health care system in India.

The findings of this study should be generalised with caution. The above discussion on the results of this study and their interpretation are subject to the limitations of the data. The data used for this study are taken from a nationally representative survey of households designed to collect data on morbidity and health care of individual members in the sampled households, not from admitted inpatients of hospitals or administrative records of the hospital. As such, data used for the analysis in this paper are subject to recall bias and digit preference. For this reason, the results and interpretations are influenced up to some extent due to reporting bias.
